# Fat Fraction Extracted from Whole-Body Magnetic Resonance (WB-MR) in Bone Metastatic Prostate Cancer: Intra- and Inter-Reader Agreement of Single-Slice and Volumetric Measurements

**DOI:** 10.3390/tomography10070075

**Published:** 2024-06-28

**Authors:** Giorgio Maria Agazzi, Nunzia Di Meo, Paolo Rondi, Chiara Saeli, Alberto Dalla Volta, Marika Vezzoli, Alfredo Berruti, Andrea Borghesi, Roberto Maroldi, Marco Ravanelli, Davide Farina

**Affiliations:** 1 Neuroradiology Department, ASST Santi Paolo e Carlo, 20142 Milan, Italy; giorgiomaria.agazzi@gmail.com; 2 Radiology Unit, Department of Medical and Surgical Specialties, Radiological Sciences and Public Health, University of Brescia, P.le Spedali Civili 1, 25123 Brescia, Italyandrea.borghesi@unibs.it (A.B.); roberto.maroldi@unibs.it (R.M.); marco.ravanelli@unibs.it (M.R.); davide.farina@unibs.it (D.F.); 3 Department of Radiology, University of Brescia, P.le Spedali Civili 1, 25123 Brescia, Italy; chiara.saeli@gmail.com; 4 Department of Oncology, University of Brescia, P.le Spedali Civili 1, 25123 Brescia, Italy; alberto.dallavolta@gmail.com (A.D.V.);; 5 Department of Molecular and Translational, University of Brescia, Piazza del Mercato 15, 25123 Brescia, Italy

**Keywords:** fat fraction, radiomics, bone metastasis, MET-RADS-P, prostate cancer, whole-body magnetic resonance imaging

## Abstract

Background: This study evaluates the repeatability and reproducibility of fat-fraction percentage (FF%) in whole-body magnetic resonance imaging (WB-MRI) of prostate cancer patients with bone metastatic hormone naive disease. Methods: Patients were selected from the database of a prospective phase-II trial. The treatment response was assessed using the METastasis Reporting and Data System for Prostate (MET-RADS-P). Two operators identified a Small Active Lesion (SAL, <10 mm) and a Large Active Lesion (LAL, ≥10 mm) per patient, performing manual segmentation of lesion volume and the largest cross-sectional area. Measurements were repeated by one operator after two weeks. Intra- and inter-reader agreements were assessed via Interclass Correlation Coefficient (ICC) on first-order radiomics features. Results: Intra-reader ICC showed high repeatability for both SAL and LAL in a single slice (SS) and volumetric (VS) measurements with values ranging from 0.897 to 0.971. Inter-reader ICC ranged from 0.641 to 0.883, indicating moderate to good reproducibility. Spearman’s rho analysis confirmed a strong correlation between SS and VS measurements for SAL (0.817) and a moderate correlation for LAL (0.649). Both intra- and inter-rater agreement exceeded 0.75 for multiple first-order features across lesion sizes. Conclusion: This study suggests that FF% measurements are reproducible, particularly for larger lesions in both SS and VS assessments.

## 1. Introduction

In recent years, whole-body magnetic resonance (WB-MR) has become a widely used technique to assess treatment response [1] in multiple myeloma, breast cancer, and prostate cancer [2,3,4].

If the integration of diffusion-weighted (DWI) sequences allows the medullary cellularity changes to be detected earlier than with morphologic sequences, the T1-weighted gradient-echo Dixon sequences generate four parameters simultaneously (in-phase, IP; out-phase, OP; fat-only, FO; water-only, WO), which describe specific characteristics of medullary bone and metastases. Combining these parameters through the equation FO/(FO + WO), it is possible to obtain the parameter named “fat fraction” (FF%), which can estimate the percentage of hemopoietic and adipose medullary bone and estimate the presence of fat within the metastases involving the medullary bone. FF% decreases in the case of malignant infiltration, while increasing as the cancer tissue is progressively replaced by fatty bone marrow restoration. It has been demonstrated that FF% can stratify early treatment response in multiple myeloma, which makes it better than DWI [5,6,7].

In multiple myeloma, the repeatability of measurements of apparent diffusion coefficient (ADC) derived from DWI has been demonstrated to be excellent even when a central single slice was contoured; conversely, for FF% both repeatability and reproducibility are influenced by lesion size [8].

Semiautomatic volumetric segmentation has been conducted in recent years with a variety of open source or proprietary software [9]. Petralia et al., using one of these software packages, have demonstrated an excellent intra- and inter-observer inter-class correlation coefficient (ICC) for ADC measurement in breast cancer and a moderate to excellent ICC in prostate cancer [10].

At the moment, as far as we know, there are no studies focused on the repeatability and reproducibility of FF% in prostate cancer patients with bone metastatic hormone naive disease. This study aims to demonstrate that and to evaluate the concordance of FF% values obtained with a single-slice and a volumetric segmentation.

## 2. Materials and Methods

### 2.1. Patient Selection

Thirty-four patients were randomly extracted from the entire list of 126 patients of the prospective phase II BonEnza trial. The number 34 was calculated using the sample-size calculation method described in the statistical analysis section. The BonEnza trial aims to test the activity of LHRH-A plus enzalutamide plus/minus zoledronic acid in terms of bone response assessed by WB-MR in prostate cancer patients with bone metastatic hormone naive disease. The local ethics committee approved the prospective phase II BonEnza trial, and written informed consent was obtained from the subjects for the use of their data. The study was written in compliance with the GRRAS checklist [11].

### 2.2. Imaging Protocol

The WB-MR examinations were performed using a 1.5T MR scanner (MAGNETOM Aera, Siemens Healthcare, Erlangen, Germany). The scanning protocol (shown in Table 1) was MET-RADS-P compliant [3].

In particular, DWI scans extended from the upper border of the orbits to mid-thigh and consisted of four contiguous stations of 50 slices acquired in free-breathing using 2D single-shot echo-planar imaging (SS-EPI). The shimming technique used for the DWI scans was Station-Specific Shim, through which a single volumetric shim was determined for each station and applied for all slices within the station. Axial T1-DIXON and T2 HASTE scans extended from vertex to mid-thigh.

FF% is extrapolated from the T1 Dixon sequence, which provides a set of four different images as follows: in-phase, out-of-phase, water only, and fat only. FF% maps were computed using the following equation:

$$FF\%=\left(\frac{fat\;only}{water\;only+fat\;only}\right)*\;100$$

A typical cumulative WB-MRI data acquisition time per examination was 50 min.

### 2.3. Image Segmentation

**Figure 1:**
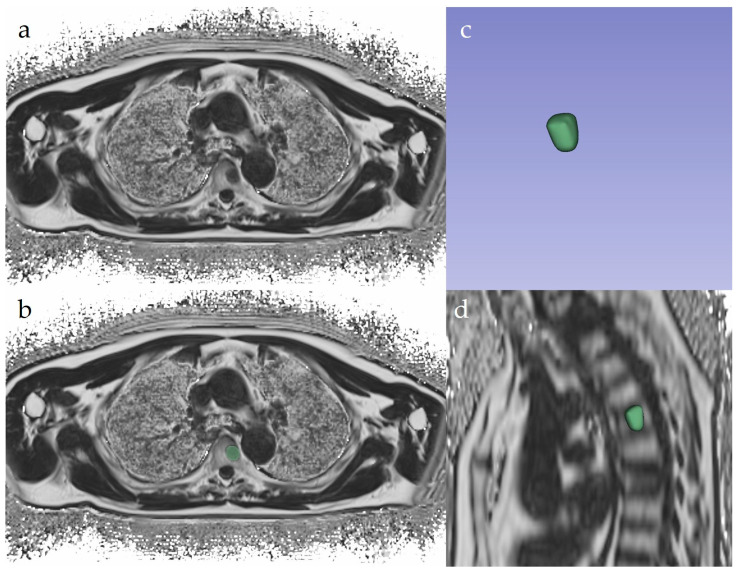
Graphic example of volumetric segmentation using 3D Slicer: (**a**) axial fat-fraction image without segmentation; (**b**) axial fat-fraction image with manual segmentation of the lesion; (**c**) volumetric rendering of the multiple slice segmentations; (**d**) volumetric segmentation displayed on a sagittal reconstruction. Volumetric segmentation was done drawing a polygonal ROI encompassing the entire lesion on every slice in which the lesion itself was visible.

For each patient, two operators in consensus identified a small lesion (<10 mm axial diameter) and a large lesion (≥10 mm axial diameter), annotating the positions of both. Lesion selection was performed by evaluating DWI images along with FF% and other morphologic sequences, to identify and segment only active focal lesions. Segmentation was performed manually and independently by the two readers: reader 1 with one year of experience and reader 2 with three years of experience in whole-body imaging. Reader 1 segmented each lesion twice to assess intra-reader agreement, and reader 2 segmented each lesion once to assess inter-reader agreement. Single-slice measurements were performed on the axial slice deemed to be the most representative of the whole lesion (i.e., the slice with the largest cross-sectional area). The choice of the most representative slice was performed independently by the two readers who were blinded to the selection of the other reader. Therefore not all the lesions were segmented on the same slice by both readers, which represents a setting similar to clinical routine. Volumetric segmentation was performed on axial sections (Figure 1). All segmentations were performed with the freeware software 3DSlicer (v.5.6.2).

### 2.4. First-Order Features Analysis

First-order features were extracted using the Python library pyradiomics (v 3.0.1) on original unfiltered images. Voxel outliers more than three standard deviations away from the mean were removed before computing features, to reduce influence related to segmentation errors or extreme values. Voxels were resampled to a dimension of 3 × 3 × 3 mm to look at a coarser texture. Intensity was rescaled in percentage value to account for fat-fraction map calculation variability (some maps were calculated with a factor of 100, others with a factor of 1000). The bin count was fixed at 64 and texture was extracted with a 2D slice-by-slice approach to account for non-isotropic sequences.

### 2.5. Statistical Analysis

Sample size calculation was made according to results published in a previous study [8].

For a repeated measure standard deviation ratio of 2 between small and large lesions, according to previous data 44 lesions are required (at least 22 patients with one small and large lesion each). Within-subject standard deviations and differences in within-subject variances were calculated according to Barwick et al. [8]. Levene’s test was performed to formally test differences in within-subject variances between small and large lesions, for both single-slice and whole-volume measurements. Inter- and intra-rater agreement of FF% were evaluated with ICC, using a one-way single-measure absolute agreement model. Barwick et al. reported excellent inter- and intra-rater agreement of axial mean FF% (ICC > 0.9) [8]. We calculated a minimum sample size of 33 patients according to 0.8 power, 0.05 statistical significance, minimum acceptable reliability of 0.75, and expected reliability of 0.9. The final sample size was 34 patients. 

Bland–Altman plots were also used for visual inspection of agreement between measurements.

Intra-reader agreement was evaluated after two weeks to minimize recall bias. Inter-reader agreement was evaluated between the mean measurements of reader 1 and the measurements of reader 2.

Agreements were calculated for both single-slice measurements and whole-volume measurements. ICC was considered to be poor if less than 0.5, moderate if between 0.5 and 0.75, good if between 0.75 and 0.9, and excellent if >0.9 [12].

Agreement analysis was performed also for first-order features on volumetric segmentations, for both small and large lesions. Inter-reader agreement for volumetric first-order features was performed by comparing the first measurement of reader 1 and the unique measurement of reader 2. We did not compute the mean of measurements of reader 1 for this analysis because we considered it inappropriate to calculate a mean for some of the first-order features extracted (such as kurtosis or skewness, which may have positive or negative values).

The correlation between single-slice measurements and whole-volume measurements for mean FF% value was evaluated using measurements of the three readers (two from reader 1 and one from reader 2) using Spearman’s correlation coefficient. The correlation was considered moderate if between 0.5 and 0.75, good if between 0.75 and 0.9, and excellent if >0.9. Spearman’s rho 95% CI was calculated with Bootstrap with one repetition.

Statistical analysis was performed with R v3.6.0.3. 

## 3. Results

A total of 34 patients were included in our study. Of these, 28 had small lesions that were large enough to be segmented on more than a single slice. The remaining patients had small lesions that segmented on a single slice only, so they were excluded from volume measurements and were included only for single-slice measurements.

### Agreement

**Figure 2:**
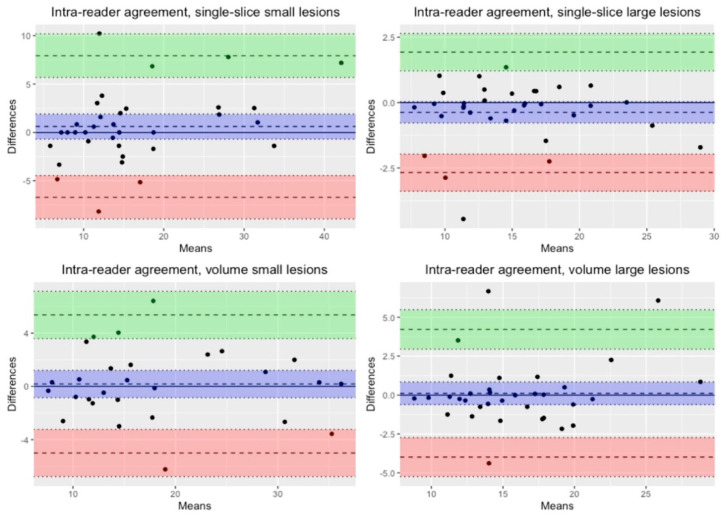
Bland–Altman plots for intra-reader agreement in small and large lesions, single-slice and hole-volume measurements. The means are expressed as fat-fraction percentages (FF%) and represent the average of the two measurements. These measurements were taken by one operator for intra-reader agreement. The differences are also expressed as percentages, representing the percentage differences between the two measurements.

**Figure 3:**
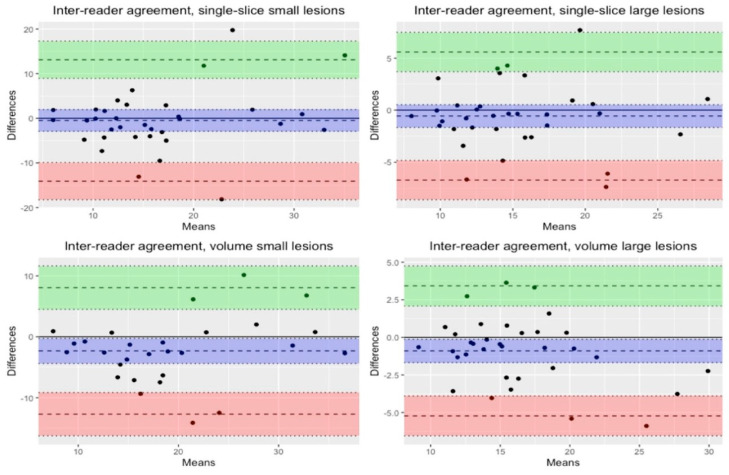
Bland–Altman plots for inter-reader agreement in small and large lesions, single-slice and whole-volume measurements. The means are expressed as fat-fraction percentages (FF%) and represent the average of the two measurements. These measurements were taken by the two different operators for inter-rater agreement. The differences are also expressed as percentages, representing the percentage differences between the two measurements.

Variances between measurements of small and large lesions were significantly different, with smaller lesions having higher measurement variances for both single slice (*p*-value < 0.001) and whole volume (*p*-value = 0.008). Visual inspection of Bland–Altman plots showed higher agreement between intra-reader measurements compared to inter-reader measurements (see Figure 2, Figure 3 and Table 2).

Summary ICC for intra- and inter-reader agreement and correlations between single-slice and volumetric measurements are reported in Table 3.

Moderate agreement was observed in inter-reader single-slice measurements for small lesions. Good agreement was observed in inter-reader volumetric measurements for small lesions, inter-reader single-slice and volumetric measurements of large lesions, and intra-reader volumetric measurements of large lesions. Excellent agreement was observed in intra-reader measurements of small lesions, and single slices.

ICCs of volumetric measurements were higher compared to ICCs of single-slice measurements for intra- and inter-reader agreement in small lesions and for inter-reader agreement in large lesions. Only intra-reader single-slice measurements had higher ICCs compared to volumetric ones. ICCs of intra-reader agreement were higher for all measurements compared to ICCs of inter-reader agreement. Given the low sample size and wide overlapping confidence intervals, we could not evaluate the statistical significance of these results.

**Figure 4:**
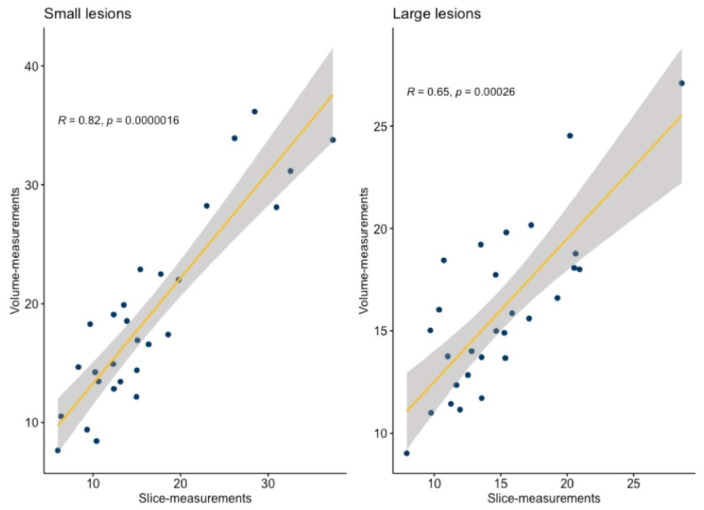
The plot shows a correlation between single-slice measurements and volumetric measurements. *R* = Spearman’s rho, yellow line = regression line, gray bands = 95% confidence bands.

The strong correlation between single-slice and volumetric measurements was observed for small lesions (Spearman’s rho 0.817), while only a moderate correlation was observed in large lesions (Spearman’s rho 0.649, see Figure 4).

First-order volumetric feature agreement was possible on 30 large lesions and 27 small lesions. Full agreement results are reported in the Supplementary Materials.

For large lesions, 10Percentile, 90Percentile, Median, Minimum, and RootMeanSquared had both inter- and intra-reader agreement > 0.75.

For small lesions, 10Percentile, Median, Minimum, and RootMeanSquared had both inter- and intra-reader agreement > 0.75. We did not compute a correlation analysis between single-slice and volumetric measurements for histogram features. 

## 4. Discussion

The development of new target drugs for treating bone metastatic castration resistant prostate cancer (mCRPC) patients led to an improvement in overall survival (OS), quality of life (QoL), and control of pain [13]. However, the response among patients is heterogeneous and the validation of biomarkers for early stratification of the likelihood of response to personalized therapy and minimizing the loss of time and economic resources is paramount [14]. FF% has been demonstrated as valuable in tracking treatment response and is a promising biomarker of treatment outcome in multiple myeloma [15].

The image analysis of this prospective study approaches the topic of lesion quantization by assessing the inter- and intra-observer repeatability of FF%.

We also investigated the correlation between single-slice measurements of mean FF%, which represents the routine clinical approach to the more accurate but time-consuming volumetric segmentation. Conflicting studies in the literature report differences in values between 2D and 3D measurements in other fields, but this difference is not always clinically relevant [16], so this kind of analysis becomes necessary when evaluating new imaging biomarkers. If a biomarker is too variable it becomes irrevocably useless when translated into the clinical practice.

The highest values of ICC have been found for intra-observer agreement (0.914 and 0.971 for small and large lesions, respectively). This is not surprising given that the radiologist’s eye tends to repeat measurements using the same internal “settings”, with low variability over time. However, it is not realistic in clinical practice that a WB-MR is serially evaluated by the same radiologist. For this reason, there must be a preference for measurements with the minimum inter-observer variability. 

The inter-observer variability for small lesions (<1 cm) is too high, so we must be cautious when evaluating small lesions and we should choose to make measurements on larger lesions whenever possible. To include fat-fraction evaluation in response criteria such as MET-RADS-P, we must take into account the influence of lesion size on the repeatability of mean measurements, so it is reasonable to choose a lesion size large enough to be robust to measurement variations.

There is higher agreement between different operators when evaluating large lesions (>1 cm), especially for volumetric measurements when lesions are segmented on multiple slices (ICC 0.883, 95% CI 0.780–0.940). This is not surprising as the larger number of voxels selected within the core of lesions mitigates the influence of the more peripheral pixels that could be erroneously included during manual segmentation. We hypothesize by manual ROI inspection that the main source of variability could be the differing inclusion by the operators of the “tumor-healthy fat marrow interface”, which in untreated patients is often sharp, with round focal lesions with low FF% surrounded by healthy marrow tissue with high FF%. Accordingly, the slice–volume correlation for these large lesions drops down to 0.649, not justifying—in our opinion—the use of a single central slice instead of the entire volume. In single-slice measurements, segmentation errors have a greater influence on mean value leading to unreliable results. Finally, it would be recommended to segment lesions with at least 1 cm of diameter, when possible, using a volumetric approach.

An optional, more robust approach, less dependent on segmentation errors and operators’ experience compared to the mean value, could be the evaluation of first-order statistic parameters [17]. Volumetric histogram analysis has been extensively investigated in the past with promising results, and a large body of literature has demonstrated its clinical utility when applied to parametric maps, especially in diffusion-weighted imaging ADC and perfusion rCBV maps [17]. Current applications, among many, include prostate cancer grading [18] and differential diagnosis of lung lesions [19]. Test–retest studies showed that percentiles have good repeatability compared to features related to intensity distribution such as skewness or kurtosis, which are often less reliable [20,21,22]. Percentiles are less influenced by outliers and also more straightforward in the interpretation of their values compared to more elaborated heterogeneity and radiomic features, but given that repeatability can be pathology specific it should ideally be performed for each specific cohort of patients [10].

Preliminary data on ADC histogram analysis showed that histogram values are more reproducible when performed on the whole volume of a lesion compared to different single-slice approaches, in particular when evaluating extreme percentiles, but the clinical impact is unknown [23]. Our preliminary data showed, in keeping with the literature, that volumetric 10th percentile, median, minimum, and root mean squared had size-independent good agreement, with high ICC values both in large and small lesions, compared to mean values which had good agreement only for large lesions. Volumetric histogram analysis in ADC measurements also seems to be independent of lesion size, so we didn’t perform a 2D–3D correlation [10]. In patients undergoing different treatments with variable fat marrow replacement over time, often in an irregular fashion, it could be hypothesized that a 3D approach would be more reasonable, but this needs further confirmation. The need to contour larger lesions could be perceived as frustrating because it is more time-demanding; however, robust region-growing or machine learning algorithms are able (or will be in the near future) to segment the entire lesion starting from a seed point placed within the lesion by the operator [24]. The future direction is almost certainly automatization of measurement. Automatic segmentation would likely improve the agreement of the calculated FF% by reducing the variability associated with operator segmentation of lesions. This study has limitations. The sample size and the number of lesions that have been evaluated are quite low. The number of observers is just two. Although the study population of 34 subjects was calculated based on the sample size, a larger number of patients would enhance the robustness of our repeatability. Furthermore, there is not a big gap in experience between the two authors, and this could influence the inter-observer variability. Given that only pre-therapy lesions have been considered, actually we do not know the expected changes in FF% in responding lesions. For this reason, is it difficult to interpret the numeric intra- and inter-observer variability (derived from Bland–Altman plots) in terms of possible clinical utility. Histogram analysis was also not possible in a few patients because of lesion characteristics, but given the low number of excluded lesions, it is unlikely that this could have influenced the results. Segmentation has been performed manually; this is a major limitation given it is conceivable that an automatic or semiautomatic approach would have improved the measurement reproducibility, with studies reporting no variability according to lesion size when performing automated methods of segmentation [10]. We also chose not to assess repeatability of radiomic features since there are still many concerns about the repeatability of features according to image preprocessing and segmentation [25].

## 5. Conclusions

In conclusion, FF% measurement is highly reproducible when considering whole lesions >10 mm. Percentile-based histogram features showed good reproducibility; however, the clinical value of their implementation is currently unknown. The use of semiautomatic segmentation is advisable for standardizing segmentations, while studies of test–retest variability could address this potentially additional source of bias.

## Figures and Tables

**Table 1:** MET-RADS-P compliant WB-MRI scanning protocol.

SEQUENCE	DWI	T1 DIXON	T2 HASTE	T1 TSE	T2 STIR
Orientation	Axial	Axial	Axial	Sagittal	Sagittal
TR	7820	6.66	700	500	5490
TE	59	2.39	98	11	65
FOV (mm)	430	430	470	380	380
Flip Angle (°)		10			
b values (s/mm^−2^)	50,800				

**Table 2:** Bias and limit of agreement (LoA) for both single-slice and volumetric measurements. Mean (%) indicates the mean value of included lesions for comparison.

	Mean (%)	Bias	95%CI	LoA [Lower-Upper]
**Small Lesions**				
Intra-reader slice	16.3	0.611	[−0.692; 1.91]	[−6.71; 7.93]
Intra-reader volume	18.2	0.175	[−0.851; 1.2]	[−5.01; 5.36]
Inter-reader slice	16.5	−0.47	[−2.89; 1.95]	[−14.1; 13.1]
Inter-reader volume	19.4	−2.32	[−4.38; −0.268]	[−12.7; 8.07]
**Large Lesions**				
Intra-reader slice	15	−0.373	[−0.783; 0.037]	[−2.68; 1.93]
Intra-reader volume	15.9	0.114	[−0.616; 0.844]	[−3.99; 4.22]
Inter-reader slice	15.2	−0.564	[−1.66; 0.532]	[−6.72; 5.59]
Inter-reader volume	16.3	−0.9	[−1.67; −0.13]	[−5.22; 3.42]

**Table 3:** Summary agreement measures of FF% measurements in small and large lesions, both single slice and volumetric. Slice–volume correlations expressed as Spearman’s rho are also reported.

Small Lesions (<10 mm)	ICC	95% CI
Intra-reader single slice	0.914	0.837–0.956
Inter-reader single slice	0.641	0.393–0.802
Intra-reader volume	0.957	0.910–0.980
Inter-reader volume	0.762	0.551–0.882
**Large Lesions (>10 mm)**	**ICC**	**95% CI**
Intra-reader single slice	0.971	0.942–0.985
Inter-reader single slice	0.805	0.647–0.897
Intra-reader volume	0.897	0.806–0.947
Inter-reader volume	0.883	0.780–0.940
**Slice–volume correlation**	**Spearman’s rho**	**95% CI**
Small lesions	0.817	0.598–0.916
Large lesions	0.649	0.342–0.852

## Data Availability

The data presented in this study are available on request from the corresponding author.

## Supplementary Materials

The following supporting information can be downloaded at: https://www.mdpi.com/article/10.3390/tomography10070075/s1, Table S1: Intra-reader agreement large lesions; Table S2: Inter-reader agreement large lesions; Table S3: Intra-reader agreement small lesions; Table S4: Inter-reader agreement small lesions.
