# Supplementary material for: Fat Fraction Extracted from Whole-Body Magnetic Resonance (WB-MR) in Bone Metastatic Prostate Cancer: Intra- and Inter-Reader Agreement of Single-Slice and Volumetric Measurements
Source: Tomography. 2024 Jun 28;10(7):1014–23. doi: 10.3390/tomography10070075 (PMC11280977; doi:10.3390/tomography10070075)
Supplement: Supplementary file 1 [file tomography-10-00075-s001.zip › tomography-3017727-supplementary.pdf]

SUPPLEMENTARY

Large lesions

Table S1. Intra-reader agreement large lesions

Param	value	CI
X10Percentile	0,99971	[0.999-1]
X90Percentile	0,99253	[0.985-0.996]
Energy	0,932412	[0.867-0.966]
Entropy	0,850896	[0.719-0.924]
InterquartileRange	0,89323	[0.794-0.946]
Kurtosis	-0,01919	[-0.357-0.325]
Maximum	0,712948	[0.492-0.848]
MeanAbsoluteDeviation	0,934006	[0.87-0.967]
Mean	0,998982	[0.998-1]
Median	0,999178	[0.998-1]
Minimum	0,987646	[0.975-0.994]
Range	0,716752	[0.498-0.85]
RobustMeanAbsoluteDeviation	0,911313	[0.828-0.956]
RootMeanSquared	0,998914	[0.998-0.999]
Skewness	0,132064	[-0.218-0.454]
TotalEnergy	0,932412	[0.867-0.966]
Uniformity	0,932541	[0.867-0.966]
Variance	0,827095	[0.677-0.911]

Table S2. Inter-reader agreement large lesions

Param	value	CI
X10Percentile	0,981129	[0.961-0.991]
X90Percentile	0,914041	[0.829-0.958]
Energy	-0,2841	[-0.578-0.077]
Entropy	-0,3805	[-0.645--0.032]
InterquartileRange	0,448527	[0.116-0.692]
Kurtosis	-0,17638	[-0.497-0.189]
Maximum	0,759882	[0.557-0.878]
MeanAbsoluteDeviation	0,470047	[0.142-0.706]
Mean	0,97068	[0.94-0.986]
Median	0,972832	[0.944-0.987]
Minimum	0,953091	[0.905-0.977]
Range	0,30657	[-0.05-0.595]
RobustMeanAbsoluteDeviation	0,367796	[0.019-0.638]
RootMeanSquared	0,969939	[0.938-0.986]
Skewness	-0,00657	[-0.357-0.347]
TotalEnergy	-0,28228	[-0.576-0.079]
Uniformity	-0,01843	[-0.367-0.337]
Variance	0,265035	[-0.095-0.565]

Small lesions

Table S3. Intra-reader agreement small lesions

Param	value	CI
X10Percentile	0,986567	[0.971-0.994]
X90Percentile	0,882621	[0.758-0.945]
Energy	0,356834	[-0.022-0.648]
Entropy	-0,02217	[-0.395-0.36]
InterquartileRange	0,702275	[0.443-0.854]
Kurtosis	-0,03842	[-0.409-0.345]
Maximum	0,471315	[0.116-0.721]
MeanAbsoluteDeviation	0,770896	[0.556-0.89]
Mean	0,971189	[0.938-0.987]
Median	0,977482	[0.951-0.99]
Minimum	0,971684	[0.939-0.987]
Range	0,529775	[0.192-0.757]
RobustMeanAbsoluteDeviation	0,688798	[0.422-0.847]
RootMeanSquared	0,970288	[0.936-0.987]
Skewness	0,112647	[-0.275-0.471]
TotalEnergy	0,382612	[0.008-0.665]
Uniformity	-0,09879	[-0.458-0.291]
Variance	0,654472	[0.369-0.828]

Table S4. Inter-reader agreement small lesions

Param	value	CI
X10Percentile	0,894071	[0.783-0.95]
X90Percentile	0,49933	[0.159-0.735]
Energy	-0,43407	[-0.692--0.074]
Entropy	-0,676	[-0.836--0.406]
InterquartileRange	0,231615	[-0.151-0.556]
Kurtosis	0,024083	[-0.348-0.392]
Maximum	0,51468	[0.179-0.744]
MeanAbsoluteDeviation	0,262853	[-0.118-0.578]
Mean	0,768937	[0.558-0.887]
Median	0,753672	[0.533-0.879]
Minimum	0,888975	[0.774-0.948]
Range	0,465815	[0.116-0.714]
RobustMeanAbsoluteDeviation	0,199012	[-0.184-0.532]
RootMeanSquared	0,763901	[0.55-0.885]
Skewness	0,057703	[-0.318-0.421]
TotalEnergy	-0,44831	[-0.702--0.092]
Uniformity	-0,06176	[-0.422-0.317]
Variance	0,039485	[-0.335-0.405]
